# Effect of *Enterococcus faecalis* OG1RF on human calvarial osteoblast apoptosis

**DOI:** 10.1186/s12903-022-02295-y

**Published:** 2022-07-08

**Authors:** Yang Li, Shuyu Sun, Cheng Wen, Jialin Zhong, Qianzhou Jiang

**Affiliations:** 1 Department of Endodontics, Affiliated Stomatology Hospital of Guangzhou Medical University, Guangdong Engineering Research Center of Oral Restoration and Reconstruction, Guangzhou Key Laboratory of Basic and Applied Research of Oral Regenerative Medicine, Guangzhou, 510182 China; 2 Department of Endodontics, Stomatological Hospital, Southern Medical University, Guangzhou, China

**Keywords:** BCL-2 family, Apoptosis, *Enterococcus faecalis*, Osteoblast, Periapical periodontitis

## Abstract

**Background:**

*Enterococcus faecalis* is a dominant pathogen in the root canals of teeth with persistent apical periodontitis (PAP), and osteoblast apoptosis contributes to imbalanced bone remodelling in PAP. Here, we investigated the effect of *E. faecalis* OG1RF on apoptosis in primary human calvarial osteoblasts. Specifically, the expression of apoptosis-related genes and the role of anti-apoptotic and pro-apoptotic members of the BCL-2 family were examined.

**Methods:**

Primary human calvarial osteoblasts were incubated with *E. faecalis* OG1RF at multiplicities of infection corresponding to infection time points. Flow cytometry, terminal deoxynucleotidyl transferase dUTP nick end labelling (TUNEL) assay, caspase-3/-8/-9 activity assay, polymerase chain reaction (PCR) array, and quantitative real-time PCR were used to assess osteoblast apoptosis.

**Results:**

*E. faecalis* infection increased the number of early- and late-phase apoptotic cells and TUNEL-positive cells, decreased the mitochondrial membrane potential (Δ*Ψ*m), and activated the caspase-3/-8/-9 pathway. Moreover, of all 84 apoptosis-related genes in the PCR array, the expression of 16 genes was upregulated and that of four genes was downregulated in the infected osteoblasts. Notably, the mRNA expression of anti-apoptotic *BCL2* was downregulated, whereas that of the pro-apoptotic *BCL2L11*, *HRK*, *BIK*, *BMF*, *NOXA*, and *BECN1* and anti-apoptotic *BCL2A1* was upregulated.

**Conclusions:**

*E. faecalis* OG1RF infection triggered apoptosis in human calvarial osteoblasts, and BCL-2 family members acted as regulators of osteoblast apoptosis. Therefore, BCL-2 family members may act as potential therapeutic targets for persistent apical periodontitis.

## Background

Persistent apical periodontitis (PAP) is an endodontic inflammatory condition characterised by bone destruction in the periapical region [1]. Inflammatory bone destruction is generally associated with an imbalance between bone degradation and formation [2]. In addition to enhanced osteoclastogenesis and subsequential bone resorption, increased osteoblast apoptosis decreases the synthesis of bone matrix and deposition of new bone, resulting in bone loss in inflammatory bone diseases [3]. Bacteria and their by-products are considered responsible for osteoblast apoptosis in osteomyelitis, periodontitis, and PAP [4–6]. Evidence has highlighted that *Enterococcus faecalis* is one of the causative microorganisms of PAP [7, 8]. The detection of *E. faecalis* in infected root canals has been associated with the occurrence of periapical lesions larger than 3 mm, indicating the role of *E. faecalis* in PAP and endodontic treatment failure [9]. Recent studies have found that *E. faecalis* induces apoptosis in mouse osteoblast-like MC3T3 cells and human osteosarcoma MG63 cells [10–12]. However, there may be a difference between the effect of *E. faecalis* infection in primary osteoblasts and osteoblast-like cells from different origins [10, 13]. The apoptosis-inducing effect of *E. faecalis* and its mechanisms in human primary osteoblasts remain elusive.

Bacteria may induce apoptosis in osteoblasts via intrinsic (caspase-9 dependent) and/or extrinsic (caspase-8 dependent) pathway(s) through the activation of executioner caspase-3 [5, 11]. Apoptosis-related genes, such as tumour necrosis factor receptor superfamily (TNFRSF) and caspase activity inhibitors (e.g., BIRC6, NAIP, and XIAP), may also play important roles in infection-induced apoptosis [14]. Notably, the B-cell lymphoma-2 (BCL-2) family plays a key role in the intrinsic apoptotic pathway [15]. The sophisticated modulation of the balance between anti-apoptotic and pro-apoptotic members of the BCL-2 family can determine cell fate decisions of life or death, giving rise to numerous diseases, such as cancer and autoimmune diseases [16, 17]. Studies have reported that anti-apoptotic BCL-2 and BCL2L1 (also known as BCLXL), and pro-apoptotic BID, BIM, and BAX may play roles in the pathogenesis of periodontitis [5, 18, 19]. BCL-2, BCL2L1, BAD, and BAX may also be involved in the molecular mechanisms linking periodontal disease with cancer [20]. However, the putative role of the BCL-2 family and other apoptosis-related genes in *E. faecalis*-infected primary osteoblasts remains inconclusive.

Here, we investigated the apoptotic effect of *E. faecalis* OG1RF on primary human calvarial osteoblasts. The strain OG1RF is a rifampicin- and fusidic acid-resistant derivative of a caries-related strain OG1 isolated in 1975 [21, 22]. Because of its oral rather than urinary origin (e.g., ATCC 29,212), it is commonly used to investigate the pathogenicity of *E. faecalis* in apical periodontitis [23, 24]. We examined the mRNA expression of 84 apoptosis-related genes, and explored the role of anti-apoptotic and pro-apoptotic members of the BCL-2 family in *E. faecalis*-induced osteoblast apoptosis.

## Methods

### *Cultures of osteoblasts and E. faecalis* OG1RF

Primary human calvarial osteoblasts were obtained from ScienCell Research Laboratories (#4600; Carlsbad, CA, USA) and cultivated in a medium (#4601) containing 1% osteoblast growth supplement (#4652), 1% penicillin/streptomycin solution (#0503), and 5% foetal bovine serum (#0025) at 37 °C in a humidified atmosphere with 5% CO_2_. In this study, cells from passages 3 to 6 were used. *E. faecalis* OG1RF, obtained from ATCC (Manassas, VA, USA), was cultured in brain–heart infusion broth (Difco Laboratories, Detroit, MI, USA) under aerobic conditions at 37 °C to the mid-logarithmic phase [22].

To examine the effect of *E. faecalis* OG1RF on osteoblast apoptosis, the cells were plated onto 24-well plates at 1.5 × 10^5^/well or six-well plates at 6 × 10^5^/well. At the indicated infection time points, *E. faecalis* OG1RF at the corresponding multiplicity of infection (MOI) was added to the osteoblast culture, as previously described [12].

### Flow cytometry

To evaluate the apoptotic rate of cells (in early and late phases), primary osteoblasts were incubated with *E. faecalis* OG1RF at MOIs of 10, 100, 500, and 1,000 for 6 and 12 h, respectively. The infected cells were incubated with staining solution containing propidium iodide (PI) and FITC Annexin V (#556,570; BD Biosciences, San Diego, CA, USA) at 25 °C for 15 min. The apoptotic cells in early (FITC Annexin V^+^/PI^−^) and late (FITC Annexin V^+^/PI^+^) phases were analysed using a FACSCalibur flow cytometer (BD Biosciences, San Diego, CA, USA).

To detect the change in mitochondrial membrane potential (ΔΨm), primary osteoblasts were incubated with *E. faecalis* OG1RF at MOIs of 10, 100, 500, and 1,000 for 6 and 12 h, respectively. The infected cells were incubated with 0.5 mL of JC-1 staining solution (#C2006; Beyotime, Shanghai, China) and evaluated using a FACSCalibur flow cytometer. The ΔΨm was estimated by calculating the ratio of red/green fluorescence intensities as previously reported [12].

### Terminal deoxynucleotidyl transferase dUTP nick end labelling (TUNEL) assay

To examine DNA damage in apoptotic cells, primary osteoblasts were infected with *E. faecalis* OG1RF at an MOI of 1,000 for 12 h. After fixation and permeabilization, the infected cells were treated with a reaction mixture containing the label and enzyme solutions (#11,684,817,910; Roche, Penzberg, Bayern, Germany) at 37 °C for 60 min to detect TUNEL-positive cells in randomly selected microscopic fields. A 50-µL label solution without TUNEL enzyme was used as the negative control to detect nonspecific labelling.

### Caspase-3/-8/-9 activity assay

To detect the activity of caspase-3/-8/-9, primary osteoblasts were infected with *E. faecalis* OG1RF at an MOI of 1,000 for 12 h. The infected cells were evaluated using caspase-3 (#C1116)/-8 (#C1152)/-9 (#C1158) activity assay kits (Beyotime) according to the manufacturer’s instructions. After the cell lysates were harvested and incubated with protease substrates at 37 °C for 6 h, the optical densities (405 nm) of the lysates were evaluated using a microplate reader (Tecan, Reading, UK).

### Human polymerase chain reaction (PCR) array

To explore the mRNA expression profile of apoptosis-related genes in apoptotic osteoblasts, primary osteoblasts were incubated with *E. faecalis* OG1RF at an MOI of 1,000 for 12 h. The Human Apoptosis RT Profile PCR Array (#PAHS-012Z; SABiosciences, Valencia, CA, USA) was employed to simultaneously evaluate the expression of 84 apoptosis-related genes in *E. faecalis*-infected osteoblasts according to the manufacturer’s instructions. A *P*-value < 0.05 with a fold change > 2 (following normalisation to housekeeping genes) was regarded as a significant upregulation or downregulation in mRNA expression.

### Quantitative real-time PCR (qRT-PCR)

To validate the results of the PCR array and further evaluate the expression of BCL-2 family members, primary osteoblasts were infected with *E. faecalis* OG1RF at an MOI of 1,000 for 12 h. Total RNA was extracted using TRIzol reagent (#15,596,018; Invitrogen, Carlsbad, CA, USA) and quantified using Nanodrop spectrophotometer (Thermo Scientific, Wilmington, DE, USA), followed by cDNA synthesis using a reverse transcription kit (#RR036A; Takara, Kyoto, Japan). qRT-PCR was performed under universal cycling conditions using SYBR Premix Ex TaqII (#RR820A; Takara, Kyoto, Japan) in a CFX96 Real-Time PCR System (Agilent Technologies, Santa Clara, CA, USA). The 2^−ΔΔCt^ method was used to determine the expression of the following genes: *BCL2*, *BCL2L11* (also known as *BIM*), *HRK*, *BIK*, *BCL2A1* (also known as *BFL1*), *CASP10*, *BMF*, *PMAIP1* (also known as *NOXA*), *BECN1*, *BAD*, *BAX*, *HUWE1* (also known as *MULE*), *BCL2L12*, *BBC3* (also known as *PUMA*), and *BOK*. Primer sequences are listed in Table 1.

**Table 1:** Primers for qRT-PCR

Gene	GenBank accession number	Forward primer (5′ → 3′)	Reverse primer (5′ → 3′)
*BCL2*	NM_000633	CGCCCTGTGGATGACTGAGTA	GGGCCGTACAGTTCCACAAAG
*BCL2L11*	NM_006538	TAAGTTCTGAGTGTGACCGAGA	GCTCTGTCTGTAGGGAGGTAGG
*HRK*	NM_003806	GCAACAGGTTGGTGAAAACCCT	ATTGGGGTGTCTGTTTCTGCAGC
*BIK*	NM_001197	GACCTGGACCCTATGGAGGAC	CCTCAGTCTGGTCGTAGATGA
*BCL2A1*	NM_004049	TCCAAAAAGAAGTGGAAAAGAATC	GCTGTCGTAGAAGTTTCTTGATGA
*CASP10*	NM_001230	TAGGATTGGTCCCCAACAAGA	GAGAAACCCTTTGTCGGGTGG
*BMF*	AY222040	GAGCCATCTCAGTGTGTGGAG	GCCAGCATTGCCATAAAAGAGTC
*NOXA*	BC032663	ACCAAGCCGGATTTGCGATT	ACTTGCACTTGTTCCTCGTGG
*BECN1*	NM_003766	CCATGCAGGTGAGCTTCGT	GAATCTGCGAGAGACACCATC
*BAD*	NM_004322	CGGAGGATGAGTGACGAGTT	GATGTGGAGCGAAGGTCACT
*BAX*	NM_004324	CCCTTTTGCTTCAGGGTTTCATCCA	CTTGAGACACTCGCTCAGCTTCTTG
*MULE*	NM_031407	TTGGACCGCTTCGATGGAATA	TGAAGTTCAACACAGCCAAGAG
*BCL2L12*	NM_138639	GAGACCGCAAGTTGAGTGGAG	GAAGGCAGCTAGGACCCTC
*PUMA*	AF354656	GCCAGATTTGTGAGACAAGAGG	CAGGCACCTAATTGGGCTC
*BOK*	AF089746	GTCTTCGCTGCGGAGATCAT	CATTCCGATATACGCTGGGAC

qRT-PCR was performed under universal cycling conditions

### Statistical analysis

All experiments were conducted in triplicate. Statistical analysis was performed using one-way analysis of variance followed by Tukey’s post hoc test using SPSS 21.0. A *P* value of < 0.05 was considered statistically significant.

## Results

### E. faecalis infection triggered early and late apoptosis in primary osteoblasts

**Fig. 1:**
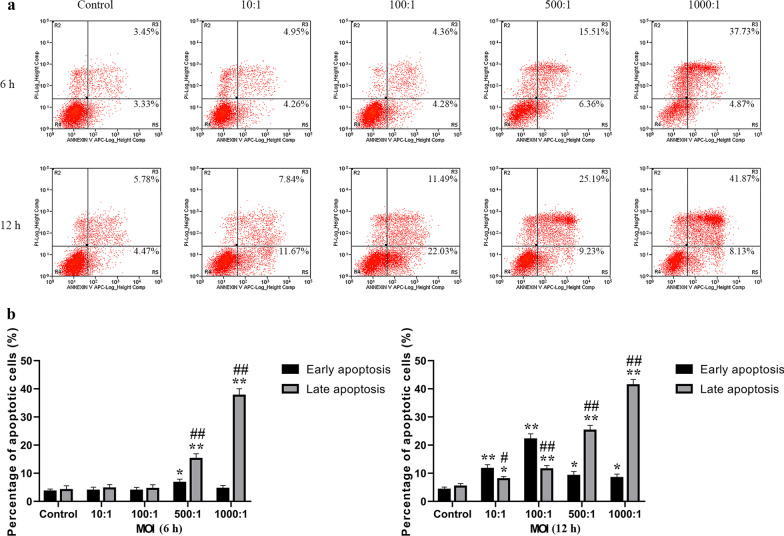
Analysis of *E. faecalis*-induced apoptotic osteoblasts in early and late phases using Annexin V-FITC/PI staining. **a** Primary osteoblasts were infected with *E. faecalis* OG1RF at MOIs of 10, 100, 500, and 1,000 for 6 and 12  h. The apoptotic cells were evaluated using flow cytometry. **b** Percentages of apoptotic cells in the early and late phases. *P < 0.05 and **P < 0.01 versus the control group. ^#^*P* < 0.05 and ^##^*P* < 0.01 versus the early apoptosis groups at the corresponding MOI and infection time point

To detect apoptosis (both in early and late phases) in osteoblasts infected with *E. faecalis* OG1RF, we conducted flow cytometry analysis using Annexin V-FITC/PI staining (Fig. 1a). Figure 1b shows the percentage of apoptotic cells in osteoblasts infected with *E. faecalis* OG1RF for 6 and 12 h. At 6 h, the peak of early apoptosis was observed at an MOI of 500 (*P* < 0.05), whereas at 12 h, it was observed at an MOI of 100 (*P* < 0.01). At 6 h, the percentage of apoptotic cells in the late phase was increased at MOIs of 500 and 1,000 compared with that in the control group (*P* < 0.01), whereas at 12 h, the increase showed an MOI-dependent trend (*P* < 0.05). Furthermore, there were more late apoptotic cells at a higher MOI (MOIs of 500 and 1,000), regardless of the infection time (6 or 12 h), and more early apoptotic cells were observed at a lower MOI (10 and 100) at 12 h (*P* < 0.05).

### E. faecalis infection caused DNA fragmentation in primary osteoblasts

**Fig. 2:**
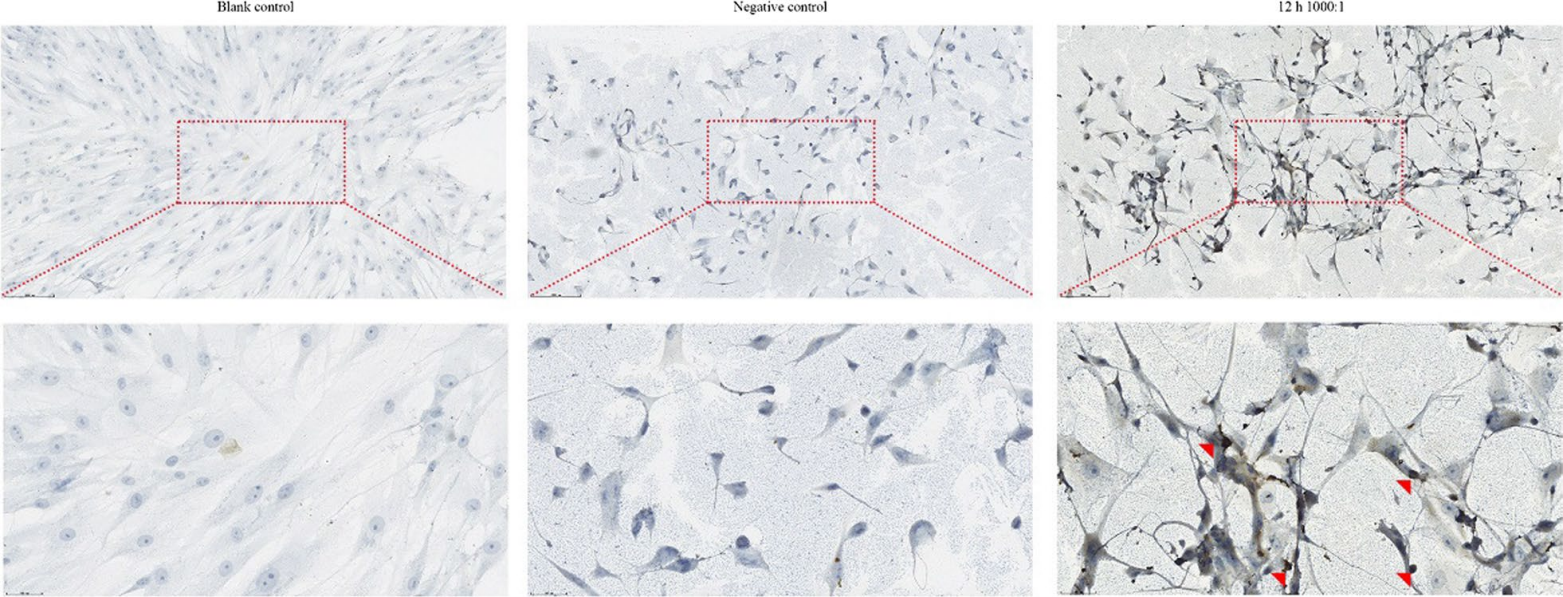
DNA fragmentation analysis in primary osteoblasts infected with *E. faecalis* OG1RF using TUNEL staining. Representative images indicating the TUNEL-positive cells (red arrows) in upper (scale bar, 200 μm) and lower (scale bar, 50 μm) panels

To examine the DNA damage in osteoblasts infected with *E. faecalis* OG1RF, we conducted TUNEL staining. TUNEL-positive cells were detected in osteoblasts following infection with an MOI of 1,000 for 12 h (Fig. 2). We observed few TUNEL-positive cells in both the negative and blank controls.

### E. faecalis infection decreased ΔΨm in primary osteoblasts

**Fig. 3:**
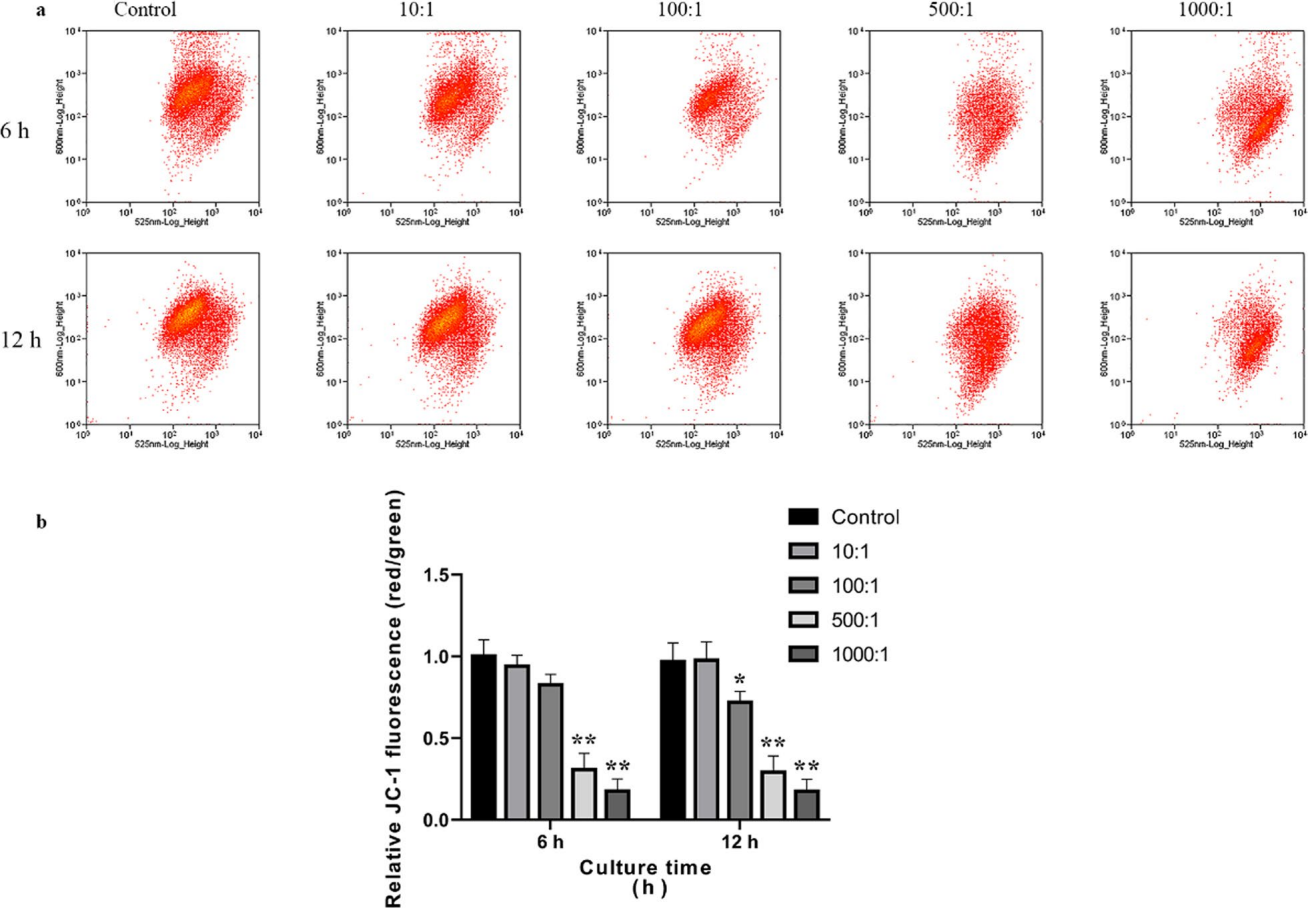
Analysis of the Δ*Ψ*m in primary osteoblasts infected with *E. faecalis* OG1RF using JC-1 staining. **a** Primary osteoblasts were incubated with *E. faecalis* OG1RF at MOIs of 10, 100, 500, and 1,000 for 6 and 12 h. The apoptotic cells were evaluated using flow cytometry after JC-1 staining. **b** The relative intensity of red/green fluorescence was determined. *P < 0.05 and **P < 0.01 versus the control group

To detect ΔΨm in osteoblasts infected with *E. faecalis* OG1RF, we conducted flow cytometry analysis using JC-1 staining (Fig. 3a). We found a significant decrease in the ratio of red/green fluorescence intensities in osteoblasts following infection with MOIs of 500 and 1,000 for 6 h and MOIs of 100, 500, and 1,000 for 12 h compared with that in the control group (*P* < 0.05), suggesting remarkably reduced ΔΨm and activated intrinsic apoptosis (Fig. 3b).

### Intrinsic and extrinsic apoptosis were involved in E. faecalis-infected osteoblasts

**Fig. 4:**
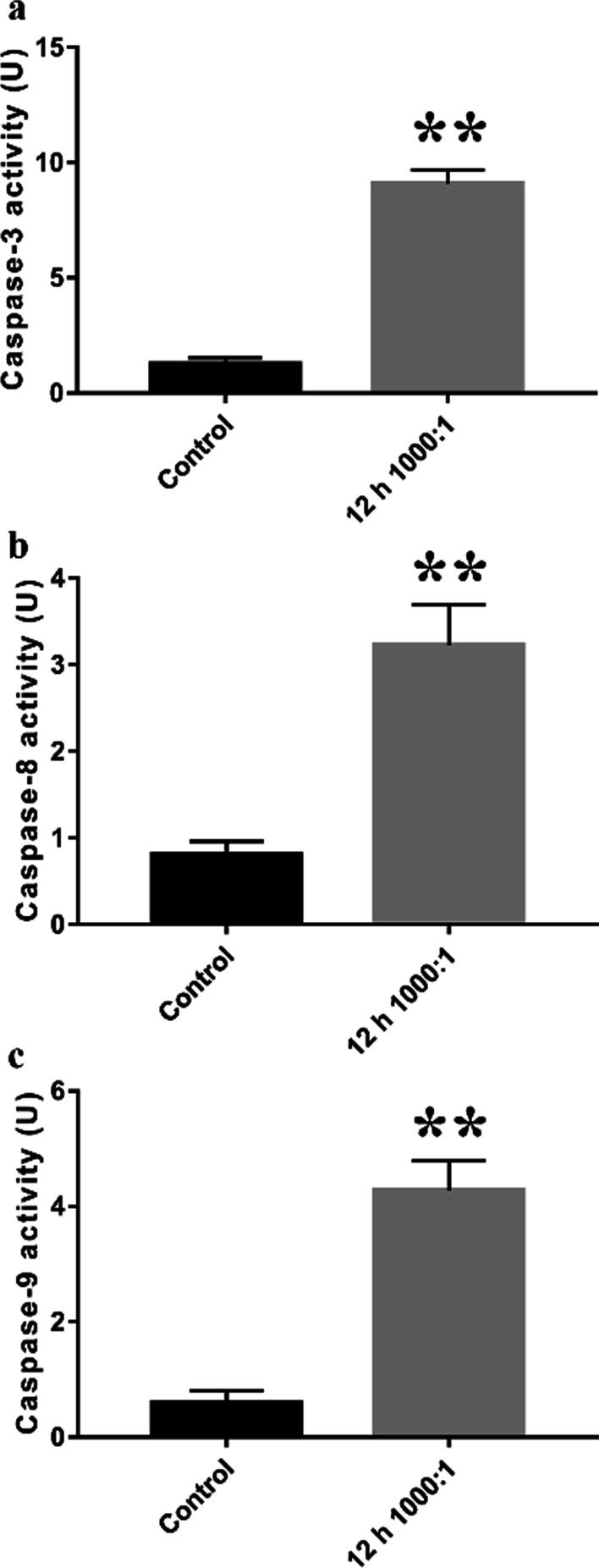
Intrinsic and extrinsic apoptotic pathways were involved in *E. faecalis-*infected osteoblasts. The activity of caspase-3 **a**, caspase-8 **b**, and caspase-9 **c** was significantly increased in the infected cells compared with the non-infected cells. **P < 0.01 versus the control group

Extrinsic and intrinsic apoptosis is initiated by activation of caspase-8 and caspase-9, respectively [25]. These activated initiator caspases can activate executioner caspase-3 to promote apoptosis [26]. To evaluate apoptosis in osteoblasts infected with *E. faecalis* OG1RF, we performed activity assays of caspase-3/-8/-9. Compared with that in the non-infected cells, the activity of caspase-3/-8/-9 was significantly increased in osteoblasts following infection at an MOI of 1000 for 12 h (*P* < 0.01), indicating that both intrinsic and extrinsic apoptotic pathways were involved. (Fig. 4).

### E. faecalis infection induced changes in mRNA expression of apoptosis-related genes in primary osteoblasts

**Fig. 5:**
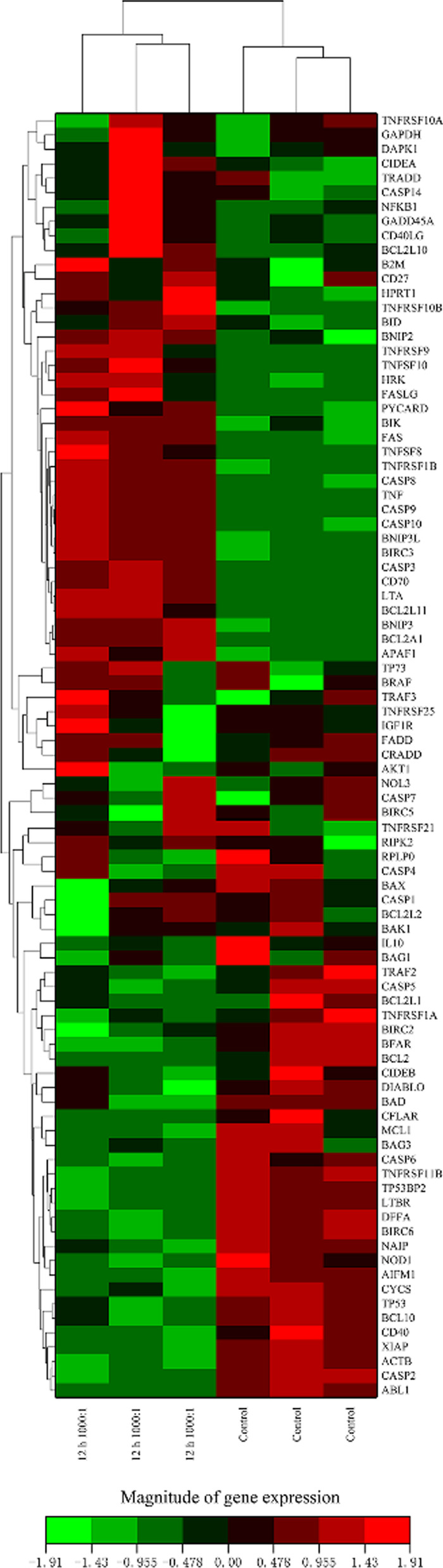
Cluster analysis of differentially expressed genes in *E. faecalis-*infected osteoblasts. Primary osteoblasts were incubated with *E. faecalis* OG1RF at an MOI of 1,000 for 12 h. Upregulated genes (red), downregulated genes (green), and unaltered genes (black) are presented in rows representing 89 genes (84 apoptosis-related and five housekeeping genes) and columns indicating the infection group (at an MOI of 1,000 for 12  h) and the control group

To identify the mRNA expression profile of apoptosis-related genes in osteoblasts infected with *E. faecalis* OG1RF at an MOI of 1,000 for 12 h, we performed PCR array analysis. Of all the 84 apoptosis-related genes in the PCR array, the expression of 16 genes was upregulated and that of four genes was downregulated in the infected cells compared with the non-infected cells, indicating the activation of apoptosis (fold change > 2 and *P* < 0.05) (Fig. 5, Table 2). Notably, within the BCL-2 family, the mRNA expression of anti-apoptotic *BCL2* was downregulated, whereas that of pro-apoptotic *BCL2L11*, *HRK*, and *BIK* and anti-apoptotic *BCL2A1* was upregulated, in the infected cells. There were no changes in the mRNA levels of *BAD*, *BID*, *BCL2L1*, *BAK*, *BAX*, *BCL2L2* (also known as *BCLW*), *BCL2L10* (also known as *BCLB*), and *MCL1* (fold change < 2 or *P* > 0.05).

**Table 2:** Apoptosis-related genes in *E. faecali*s-infected osteoblasts compared to the control sample

Rank	Gene	Accession number	Description	Fold change
*Upregulated*				
1	*TNF*	NM_000594	Tumour necrosis factor	239.50**
2	*TNFRSF9*	NM_001561	Tumour necrosis factor receptor superfamily, member 9	30.73*
3	*LTA*	NM_000595	Lymphotoxin alpha (TNF superfamily, member 1)	29.13**
4	*CD70*	NM_001252	CD70 molecule	24.76**
5	*BCL2A1*	NM_004049	BCL2-related protein A1	14.92**
6	*CASP10*	NM_001230	Caspase 10, apoptosis-related cysteine peptidase	11.04**
7	*TNFRSF10*	NM_003810	Tumour necrosis factor (ligand) superfamily, member 10	10.52*
8	*TNFRSF8*	NM_001244	Tumour necrosis factor (ligand) superfamily, member 8	10.19**
9	*BCL2L11*	NM_006538	BCL2-like 11	6.78**
10	*CASP3*	NM_004346	Caspase 3, apoptosis-related cysteine peptidase	3.40**
11	*HRK*	NM_003806	Harakiri, BCL2-interacting protein	2.64*
12	*CASP9*	NM_001229	Caspase 9, apoptosis-related cysteine peptidase	2.61**
13	*CASP8*	NM_001228	Caspase 8, apoptosis-related cysteine peptidase	2.57**
14	*TNFRSF1B*	NM_001066	Tumour necrosis factor receptor superfamily, member 1B	2.41**
15	*BIK*	NM_001197	BCL2-interacting killer	2.20**
16	*APAF1*	NM_001160	Apoptotic peptidase activating factor 1	2.21**
*Downregulated*				
1	*BCL2*	NM_000633	B-cell CLL/lymphoma 2	0.42*
2	*NAIP*	NM_004536	NLR family, apoptosis inhibitory protein	0.41**
3	*BIRC6*	NM_016252	Baculoviral IAP repeat containing 6	0.36**
4	*XIAP*	NM_001167	X-linked inhibitor of apoptosis	0.30**

Primary osteoblasts were incubated with *E. faecalis* OG1RF at an MOI of 1,000 for 12  h. Apoptosis-related genes (fold change > 2, *P* < 0.05). *P < 0.05 and **P < 0.01 versus the control group

### Validation of the expression of apoptosis-related genes in osteoblasts infected with E. faecalis

**Fig. 6:**
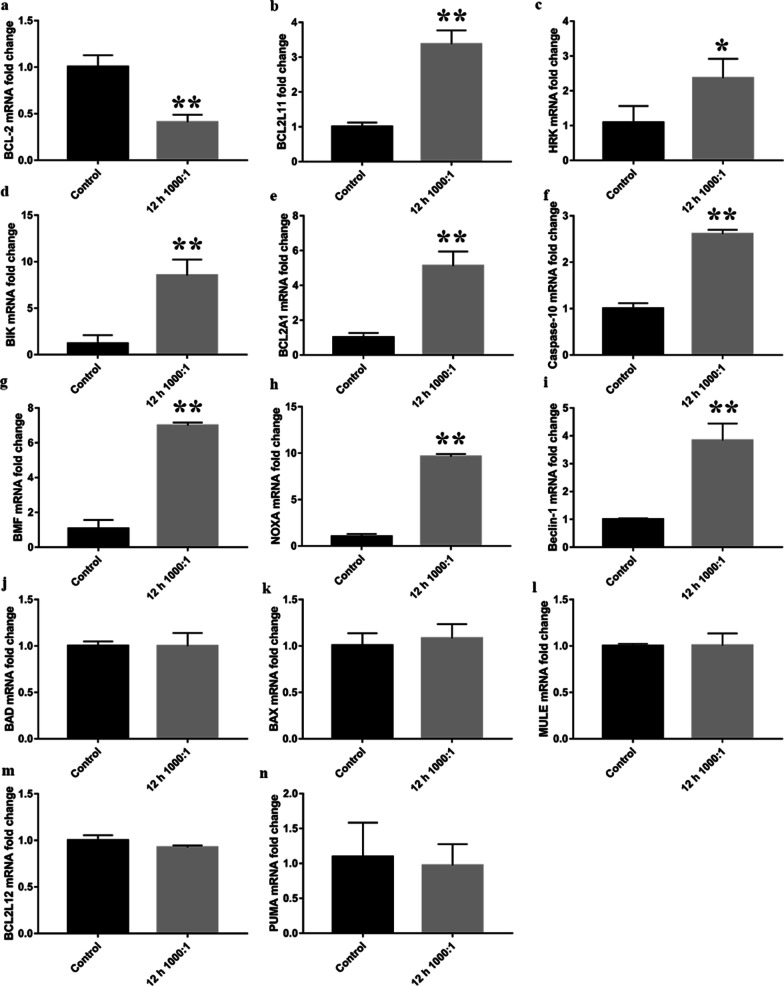
qRT-PCR analyses of the primary osteoblasts infected with *E. faecalis* OG1RF. The mRNA expression of *BCL2* **a** was downregulated, whereas that of *BCL2L11* **b**, *HRK* **c**, *BIK* **d**, *BCL2A1* **e**, *CASP10* **f**, *BMF* **g**, *NOXA* **h**, and *BECN1* **i** was upregulated, in the infected cells at an MOI of 1,000 at 12 h. The mRNA expression of *BAD* **j**, *BAX* **k**, *MULE* **l**, *BCL2L12* **m**, and *PUMA* **n** was not changed. *P < 0.05 and **P < 0.01 versus the control group

To validate the results of the PCR array and further evaluate the expression of other BCL-2 family members, we performed qRT-PCR. The mRNA expression of *BCL2* was decreased, whereas that of *BCL2L11*, *HRK*, *BIK*, *BCL2A1*, and *CASP10* was increased, in osteoblasts infected at an MOI of 1,000 for 12 h (*P* < 0.05). The expression of *BAD* and *BAX* did not change, confirming the results of the PCR array analysis. In addition, the expression of pro-apoptotic *BMF*, *NOXA*, and *BECN1* was upregulated, whereas that of *PUMA*, *MULE*, and *BCL2L12* was not altered (Fig. 6a–n). *BOK* expression was not detected.

## Discussion

PAP, a common endodontic disease induced by bacterial infection and consequent inflammatory responses, is characterised by persistent bone destruction in the periapical region [1]. Recent studies have shown that PAP lesion is associated with osteoblast apoptosis, suggesting a role of osteoblast apoptosis in the pathogenesis of PAP [27, 28]. *E. faecalis*, as one of the primary pathogens in PAP, has been reported to be highly associated with endodontic infection, promoting the disruption of bone homeostasis [9]. Hence, it is crucial to examine the role of *E. faecalis* on osteoblast apoptosis in PAP. Our previous study found that *E. faecalis* strains from the root canals of teeth with PAP trigger apoptosis in mouse MC3TE-E1 and human MG63 cell lines [10, 12]. However, the effect of *E. faecalis* on apoptosis in human primary osteoblasts and its mechanisms remain unclear.

In the present study, *E. faecalis* infection increased the number of TUNEL-positive cells and apoptotic cells in the early and late phases, which was also observed in our previous study on MC3T3-E1 and MG63 cells infected with *E. faecalis* [10, 12]. We also noticed that necrotic cells of which the cell membrane integrity is damaged may also be stained as Annexin V-FITC/PI double-positive, as previous studies reported [29, 30]. In this study, we observed that *E. faecalis* infection with a higher MOI can trigger more late apoptotic cells (and necrotic cells, if any), whereas a lower MOI infection induced more early apoptotic cells, suggesting that *E. faecalis* infection causes osteoblast apoptosis (and necrosis, if any) in an MOI-dependent way. Additionally, the mRNA expression level and activity of caspase-3/-8/-9 were elevated in the infected osteoblast group, indicating that both intrinsic and extrinsic apoptosis were activated in the osteoblasts infected with *E. faecalis* OG1RF. More precisely, the significantly decreased ΔΨm and increased expression of apoptotic peptidase activating factor 1 (APAF1) indicated the activation of intrinsic apoptosis, whereas the enhanced expression of caspase-10, which is a homologue of caspase-8, indicated the activation of the extrinsic pathway [31]. Moreover, the upregulation of TNFRSF1B, TNFRSF8, TNFRSF9, and TNFRSF10 expression, as well as elevated levels of TNF, suggested apoptotic signal transition through the TNF family membrane receptors, which was also detected in tumour cells treated with chemotherapeutic drugs [32, 33]. Furthermore, the expression of caspase inhibitors NAIP, BIRC6, and XIAP was also downregulated, indicating the promotion of apoptosis. Taken all together, these data demonstrated that both intrinsic and extrinsic apoptosis were involved in human primary osteoblasts infected with *E. faecalis*.

To further explore the activation of the intrinsic apoptotic pathway, we analysed the expression profile of the BCL-2 family in *E. faecalis*-infected osteoblasts. The BCL-2 family has two subfamilies: anti-apoptotic and pro-apoptotic [34]. The anti-apoptotic members BCL-2, BCL2L1, BCL2L2, BCL2L10, BCL2A1, BCL2L12, and MCL1 exert their function via the sequestration of the pro-apoptotic members. The pro-apoptotic subfamily is further categorised into multi-domain executioners (BAK, BAX, and BOK) and the BH3-only proteins that possess only the BCL-2 homology (BH) 3 domain. The BH3-only activators, BCL2L11, BID, PUMA, and MULE, interact with both pro-apoptotic executioners and anti-apoptotic members to trigger apoptosis, whereas BH3-only sensitisers (BAD, BMF, HRK, NOXA, BIK, and Beclin-1) displace the BH3-only activators and executioners from the anti-apoptotic protein heteromeric complex to promote apoptosis [35]. The interplay between the BCL-2 family members triggers the multi-domain executioner oligomerisation on the mitochondrial membrane, resulting in mitochondrial outer membrane permeabilization (MOMP) and subsequent activation of the caspase cascade [36]. Here, we found that the mRNA expression of anti-apoptotic *BCL2* was downregulated, whereas that of pro-apoptotic *BCL2L11*, *HRK*, *BIK*, *BMF*, *NOXA*, and *BECN1* was upregulated in the infected cells. Downregulated *BCL2* expression was also detected in MC3T3-E1 cells infected with *E. faecalis* [10]. Moreover, it has also been reported that the activator BCL2L11 and sensitisers Beclin-1 and BIK participate in osteoblast-like cell apoptosis induced by glucocorticoids, sodium fluoride, oxidative stress, and chemotherapeutic drugs [37–40]. Furthermore, decreased expression of sensitisers BMF and NOXA may increase osteoclast survival to promote bone loss [41, 42]. However, the role of harakiri (HRK), a novel regulator of cell death, in bone homeostasis remains unclear. Evidence has shown that the inactivation of HRK in prostate cancer constitutes a critical component in decreased apoptosis of tumour cells, whereas HRK-mediated mitochondrial dysfunction contributes to enhanced apoptosis in human malignancies [43]. However, in lipopolysaccharide-stimulated osteoclasts, HRK expression is not altered [41]. In this study, we reported an increase in HRK expression in the infected osteoblasts, suggesting its role in bone remodelling in PAP. In addition, interestingly, we observed that the expression of anti-apoptotic BCL2A1 was significantly higher in the infected osteoblasts than in the control sample. The pro-survival potential of BCL2A1 is reported to be associated with its interaction with BCL2L11, BIK, HRK, NOXA, BID, and PUMA [44]. The level of BCL2A1 is enhanced in different types of cancer cells, resulting in tumour progression and chemotherapy resistance [45]. *Porphyromonas gingivalis* infection also induces the upregulation of BCL2A1 in epithelial cells [46]. In this study, as the mRNA levels of *BCL2L11*, *BIK*, *HRK*, and *NOXA* were increased, we inferred that the elevated *BCL2A1* expression may act as a negative feedback factor that interacts with these four aforementioned BH3-only proteins to protect the infected osteoblasts from apoptosis. Evidently, our data suggested that these BCL-2 family members play important roles in *E. faecalis*-induced osteoblast apoptosis. Further studies are needed to elucidate the exact role and mechanism of the interaction of BCL-2 family members in the pathogenesis of PAP and their possible role(s) in the relationship of PAP with system diseases.

## Conclusions

**Fig. 7:**
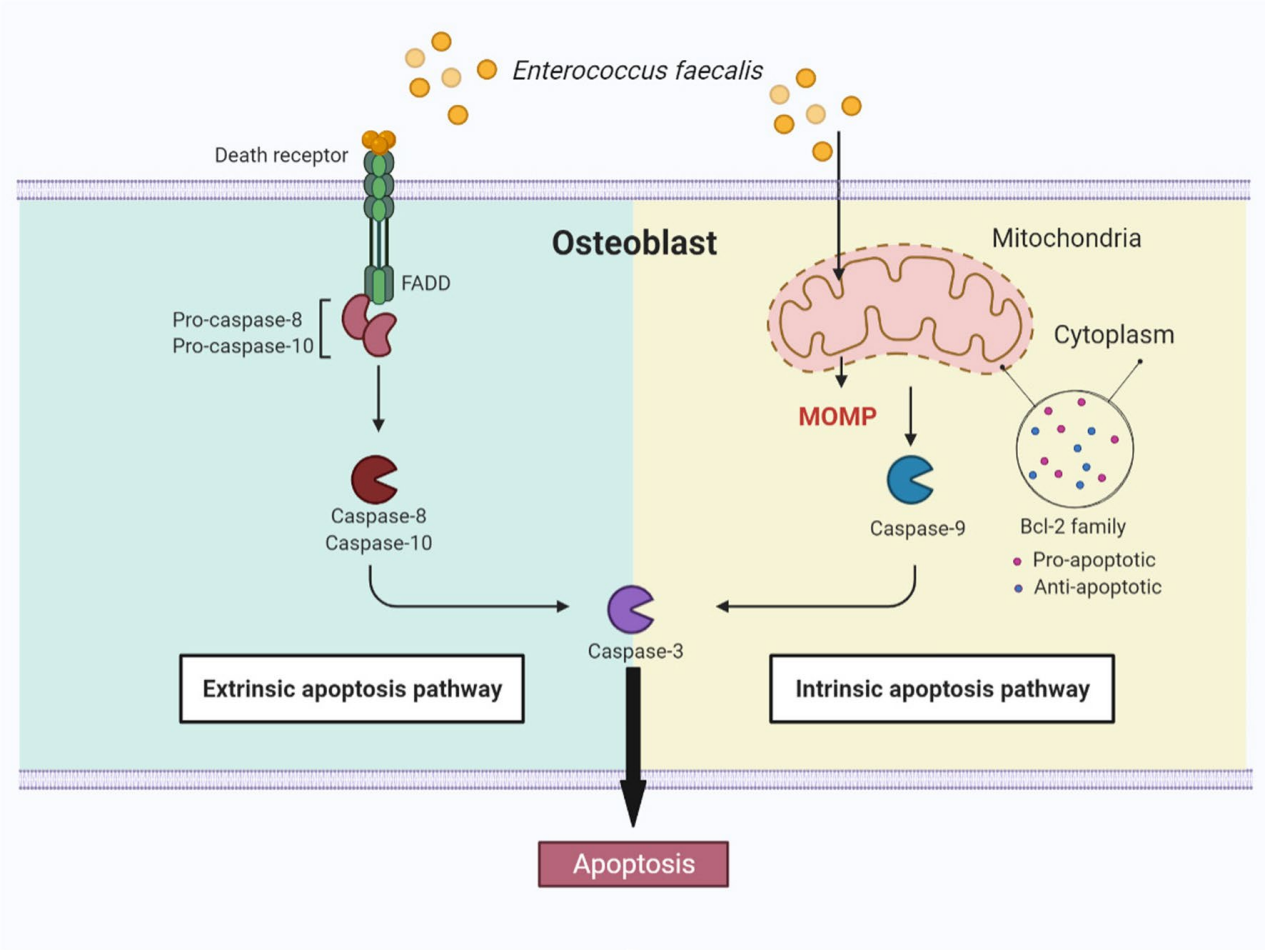
A proposed model of the mechanism of *E. faecalis*-induced-apoptosis in osteoblasts. *E. faecalis* OG1RF induced apoptosis in human primary osteoblasts, and both the extrinsic and intrinsic caspases were activated. Anti-apoptotic BCL-2 and BCL2A1, and pro-apoptotic BCL2L11, HRK, BIK, BMF, NOXA, and Beclin-1 were also involved in osteoblast apoptosis induced by *E. faecalis*

In conclusion, our findings demonstrated that *E. faecalis* OG1RF induced apoptosis in human primary osteoblasts, and both the extrinsic and intrinsic pathways were involved. Anti-apoptotic BCL-2 and BCL2A1, and pro-apoptotic BCL2L11, HRK, BIK, BMF, NOXA, and Beclin-1 were also involved in osteoblast apoptosis induced by *E. faecalis* (Fig. 7). These results suggest that BCL-2 family members act as novel regulators of osteoblast apoptosis induced by *E. faecalis* and are, thus, potential therapeutic targets for PAP treatment.

## Data Availability

The datasets generated and/or analysed during the current study are not publicly available due to data subject to third party restrictions but are available from the corresponding author on reasonable request.

## Abbreviations

APAF1: Apoptotic peptidase activating factor 1
BAX: Apoptosis regulator BAX
BCL-2: B-cell lymphoma-2
BCL2A1: BCL2-related protein A1
BCL2A1: BCL-2-related protein A1
BCL2L11: BCL2-like 11
BCL2L12: BCL-2-like protein 12
BECN1: Beclin-1
BIK: BCL2-interacting killer
BMF: BCL-2-modifying factor
BOK: BCL-2-related ovarian killer protein
HRK: Harakiri, BCL2-interacting protein
MOI: Multiplicity of infection
MOMP: Mitochondrial outer membrane permeabilization
MULE: E3 ubiquitin-protein ligase HUWE1
NOXA: Phorbol-12-myristate-13-acetate-induced protein 1
PCR: Polymerase chain reaction
PI: Propidium iodide
PUMA: BCL-2-binding component 3, isoforms 1/2
qRT-PCR: Quantitative real-time PCR
TNF: Tumour necrosis factor
TNFRSF: Tumour necrosis factor receptor superfamily
TUNEL: Terminal deoxynucleotidyl transferase dUTP nick end labelling
Δ*Ψ*m: Mitochondrial membrane potential
